# Surveillance, Epidemiology and Impact of EV-A71 Vaccination on Hand, Foot, and Mouth Disease in Nanchang, China, 2010–2019

**DOI:** 10.3389/fmicb.2021.811553

**Published:** 2022-01-06

**Authors:** Fenglan He, Jia Rui, Zhiqiang Deng, Yanxia Zhang, Ke Qian, Chunhui Zhu, Shanshan Yu, Junling Tu, Wen Xia, Qingxiong Zhu, Shengen Chen, Tianmu Chen, Xianfeng Zhou

**Affiliations:** ^1^The Collaboration Unit for Field Epidemiology of State Key Laboratory for Infectious Disease Prevention and Control, Nanchang Center for Disease Control and Prevention, Nanchang, China; ^2^State Key Laboratory of Molecular Vaccinology and Molecular Diagnostics, School of Public Health, Xiamen University, Xiamen, China; ^3^Department of Infectious Diseases, Jiangxi Provincial Children’s Hospital, Nanchang, China; ^4^Department of Pediatrics, Jiangxi Maternal and Child Health Hospital, Nanchang, China

**Keywords:** HFMD, surveillance, epidemiology, EV-A71 vaccine, Coxsackievirus A6, phylogenetic analysis

## Abstract

After the first national-scale outbreak of Hand, foot, and mouth disease (HFMD) in China, a national surveillance network was established. Here we described the epidemiology and pathogenic profile of HFMD and the impact of EV-A71 vaccination on pathogen spectrum of enteroviruses in the southeastern Chinese city of Nanchang during 2010–2019. A total of 7,951 HFMD cases from sentinel hospitals were included, of which 4,800 EV-positive cases (60.4%) were identified by real-time RT-PCR. During 2010–2012, enterovirus 71 (EV-A71) was the main causative agent of HFMD, causing 63.1% of cases, followed by 19.3% cases associated with coxsackievirus A16 (CV-A16). Since 2013, the proportion of other enteroviruses has increased dramatically, with the sub genotype D3 strain of Coxsackievirus A6 (CV-A6) replacing the dominance of EV-A71. These genetically diverse native strains of CV-A6 have co-transmitted and co-evolved in Nanchang. Unlike EV-A71 and CV-A16, most CV-A6 infections were concentrated in autumn and winter. The incidence of EV-A71 infection negatively correlated with EV-A71 vaccination (*r* = −0.990, *p* = 0.01). And severe cases sharply declined as the promotion of EV-A71 vaccines. After 2-year implementation of EV-A71 vaccination, EV-A71 is no longer detected from the reported HFMD cases in Nanchang. In conclusion, EV-A71 vaccination changed the pattern of HFMD epidemic, and CV-A6 replaced the dominance of EV-A71 over time.

## Introduction

Hand, foot, and mouth disease (HFMD) is a highly contagious disease in children caused by several human enteroviruses (EV), particularly enterovirus A71 (EV-A71) and coxsackievirus A16 (CV-A16) (Xing et al., 2014). Enteroviruses belong to the family *Picornaviridae*, genus *Enterovirus*. Their positive single-strand RNA genome is about 7,500 nucleotides, and is composed of a large open reading frame (ORF) flanked by 5′ and 3′ untranslated regions (UTRs). The 5′ part of the ORF encodes the structural proteins that form the capsid (among them the VP1 which is the most external), while the 3′ part of the ORF encodes the non-structural proteins (Palacios and Oberste, 2005). HFMD primarily affects children younger than age 5 but can infect teenagers (Xing et al., 2014; Zhou et al., 2015). Clinical manifestations of HFMD include mild to severe rash, pulmonary edema, circulatory disturbances, meningitis, aseptic encephalitis, and even death (Wang et al., 1999; Solomon et al., 2010). The severity of incidence and mortality rate of HFMD in China is about 1.1 and 0.03%, respectively (Xing et al., 2014). Since 1997, there has been outbreak of HFMD in the Asia-Pacific region, and it is a common epidemic disease worldwide. In the past decade, large outbreaks of HFMD have occurred in China, and have become a significant public health issue (Liu et al., 2011, 2014; Zong et al., 2011; Gao et al., 2014; Yin et al., 2014; Feng et al., 2015; Zhou et al., 2015). To control enteroviral infections and reduce the mortality rate of HFMD, the Ministry of Health of China has classified HFMD as a category C notifiable infectious disease in 2008 (Xing et al., 2014). Since then, an etiologic surveillance network has been established to monitor this disease. Since 2012, outbreaks of other EVs, such as Coxsackievirus A6 (CV-A6) and Coxsackievirus A10 (CV-A10), have been frequently reported in Europe, Japan and some developed regions of China. Although outbreaks of HFMD associated with CV-A6 have occurred in China, the epidemiological and etiologic characteristics of most inland cities remain unclear.

Since September 2009, the Nanchang Center for Disease Control and Prevention (NCCDC), a laboratory of the HFMD surveillance network, has initiated etiology surveillance of enteroviruses. In this study, we retrospectively analyzed the epidemiological characteristics and pathogenic profile of HFMD from 2010 to 2019, involving the inactivated EV-A71 vaccine licensed in China in December 2015 (Li et al., 2014). In 2016, the vaccine was gradually available in medical centers in China, targeting children aged 6–59 months. For optimal efficacy, two doses starting at 6 months of age, 1 month apart, are encouraged. In Nanchang, EV-A71 vaccine has been available since June 2016 and has since been termed as Category 2 vaccine, that is, the parents have to pay out of the pocket for the immunization. After the implementation of EV-A71 vaccination, the virological typing of enteroviruses expand to include CV-A6 and CV-A10 in Nanchang because of the frequent detection of CV-A6 and CV-A10 from samples of untyped enteroviruses in many developed cities of China (Tan et al., 2015; Anh et al., 2018; Horsten et al., 2018). The full-length VP1 genes of CV-A6 and CV-A10 were sequenced for representative samples and a phylogenetic tree was constructed. In addition, we observed for the first time the actual effect of EV-A71 vaccine on containing EV-A71 based on systematic surveillance of HFMD in Nanchang.

## Materials and Methods

### Data Collection

**FIGURE 1:**
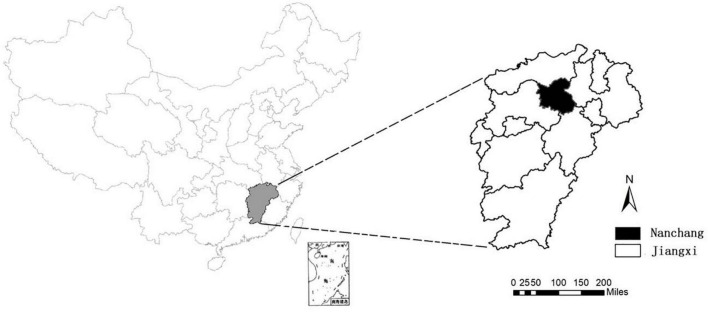
Geographical location of Nanchang City, China.

Nanchang, a city located at 115°27′–116°35′ E longitude and 28°10′–29°11′ N latitude (Figure 1). Nanchang had a population of 5.5 million as of 2019, accounting for 11.8% of the population of Jiangxi Province. Since June 2009, clinical specimens must be collected from all severe HFMD cases, and the first 5 mild cases reported per month in each county or district are tested for enteroviruses by PCR by the local CDCs (Xing et al., 2014; Li et al., 2018). Test results were characterized as enterovirus negative or positive for EV-A71, CV-A16, or other enteroviruses. Specimens positive for other enteroviruses are not routinely subjected to further serotyping. However, two more viruses, CV-A6 and CV-A10, were further detected as proportion of other enteroviruses expanded since 2015.

We collected a total of 7,951 clinically diagnosed HFMD cases from January 2010 to December 2019 from 10 hospitals in Nanchang, including Jiangxi Children’s Hospital and 9 county and district sentinel hospitals. A total of 8,188 clinical specimens (7,819 pharyngeal swabs, 126 anal swabs, 75 stools and 28 capsular swabs) were collected from 7,951 HFMD cases.

The study was approved by the ethics committee of the Nanchang Center for Disease Control and Prevention, and the procedures were performed according to the approved guidelines (Approval No. NCCDC-20100701). Prior informed consent was obtained from patients or their parents for sample collection.

### Diagnosis Criteria

Patients with the following symptoms were defined as confirmed cases of HFMD: fever; papules and herpes on the mucous membranes of the hands, feet, or mouth; rash on the buttocks or knees with inflammatory flushing around the rash, and blisters with a small amount of fluid. Severe cases of HFMD were defined as the presence of additional neurologic, cardiogenic, or pulmonary disease.

### Serotype Identification of Enteroviruses

Swabs were stored in a dedicated Universal Transport Medium (UTM) (Yocon, Beijing, China) for transport. Fecal samples were diluted to a 10% suspension using MEMs. After thorough mixing, 200 μl of clinical samples were measured for RNA extraction using the QIAamp Viral RNA Mini Kit (Qiagen, CA) according to the manufacturer’s instructions. EV, EV-A71, CV-A16, CV-A6, and CV-A10 were confirmed using a commercial real-time RT-PCR Kit (BioPerfectus technologies, Jiangsu, China) (Chen et al., 2014; Shen et al., 2018).

### Determination of the Entire VP1 Nucleotide Sequence of Coxsackievirus A6 and Coxsackievirus A10

The entire VP1 gene of CV-A6 and CV-A10 was amplified using specific primers: CV-A6 VP1-F: 5′- AACTTYGTRGTGCCACCA GA-3′(nucleotides 2,317–2,336) and CV- A 6 VP1-R: 5′-GTGGC GAGATGTCGGTTTA-3′(nucleotides 3,408–3,426) (Tan et al., 2015). CV-A10 VP1-F: 5′-CGRTAYTACACACARTGGTC-3′ (nucleotides 2,021–2,040) and CV-A10 VP1-R: 5′-CTRTCYT CCCAKACHAGGTT- 3′ (nucleotides 3,413–3,432). The whole procedure has been described previously (Ji H. et al., 2019). PCR products were purified using the QIAquick PCR Purification Kit (Qiagen, Germany), and then amplicons were sequenced in both directions using the ABI 3730 Genetic Analyzer (Applied Biosystems, United States).

### Phylogenetic Analysis and Genotyping

The VP1 sequences of CV-A6 in GenBank were first filtered to exclude laboratory-adaptive and high-pass strains and clones, and we eventually retrieved 41 entire CV-A6 VP1 full sequences from the GenBank database (Supplementary Table 1). Combined with the 106 VP1 full-length nucleotide sequences of CV-A6 determined in this study, a total of 147 sequences constituted the dataset used for genotyping. The sequences were aligned using the Clustal X program. Phylogenetic trees were constructed by the neighbor-joining method after estimation of genetic distance using the Kimura two-parameter method. A bootstrapping test was performed 1,000 times.

### Data Collection for Enterovirus 71 Vaccination

EV-A71 vaccination was initiated in Nanchang in June 2016, and vaccination records were collected from a total of 141 vaccination clinics around the study area. Children who received one dose of EV-A71 vaccine were included as the vaccinated population. Vaccination records of two doses were retained for each child who had received at least one dose.

**FIGURE 2:**
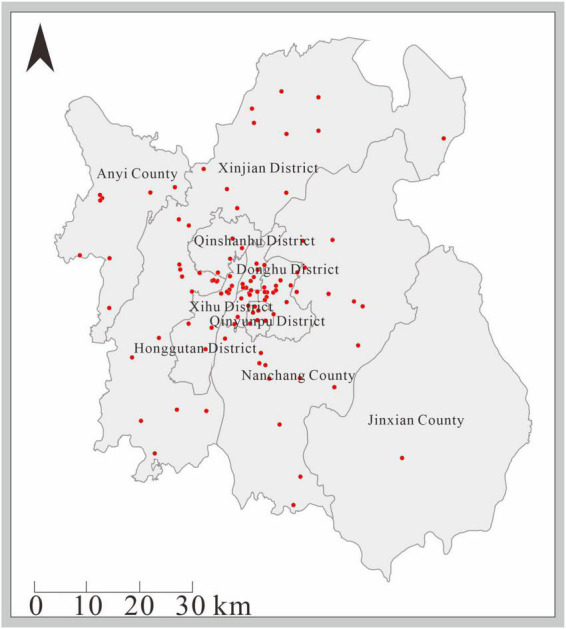
Distribution of EV-A71 vaccination sites in Nanchang City.

Vaccination clinics for HFMD were concentrated in several central urban areas (Donghu District, Qingshanhu District, Xihu District, Qingpuyun District), and there were relatively few in other surrounding areas (Figure 2).

### Statistical Analysis

We performed all statistical analysis including descriptive analysis and Pearson’s chi-square test with IBM SPSS Statistics for Windows, version 26.0 (IBM Corp., Armonk, N.Y., United States), and the difference was statistically significant with a *P-*value of < 0.05.

## Results

### General Information on Cases of Hand, Foot, and Mouth Disease (2010–2019)

**FIGURE 3:**
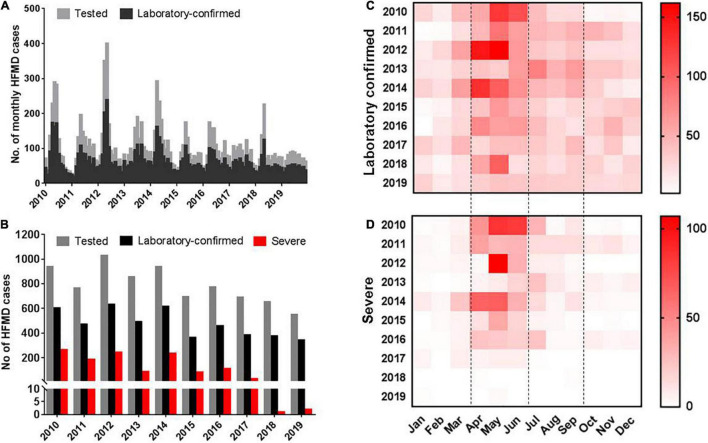
Distribution of enterovirus-associated HFMD cases in Nanchang, China from 2010 to 2019. **(A)** Monthly distribution of tested and laboratory confirmed HFMD cases. **(B)** Yearly distribution of tested, laboratory confirmed and severe HFMD cases. **(C)** Monthly heatmap of laboratory confirmed cases. **(D)** Monthly heatmap of severe HFMD cases.

From January 2010 to December 2019, a total of 7,951 suspected HFMD cases were reported to the surveillance system by sentinel hospitals in Nanchang City. The monthly distribution of detected and laboratory-confirmed HFMD (EV-positive) cases during this period were shown in Figure 3A. A larger biennial outbreak pattern was observed throughout the surveillance period. 2010, 2012, and 2014 had the highest number of reported HFMD cases. Our previous study found that subtype C4a of EV-A71 was the predominant causative agent of HFMD in children in Nanchang (Zhou et al., 2015). Since 2015, the HFMD epidemic has been generally stable, fluctuating in a lower prevalence (Figures 3A,B). In general, the incidence of HFMD peaked in the second quarter of each year, as shown by the monthly distribution of HFMD cases (Figure 3C). After 2016, the incidence of severe cases gradually decreased over time (Figures 3B,D).

### Epidemiology of Hand, Foot, and Mouth Disease in Nanchang From 2010 to 2019

**FIGURE 4:**
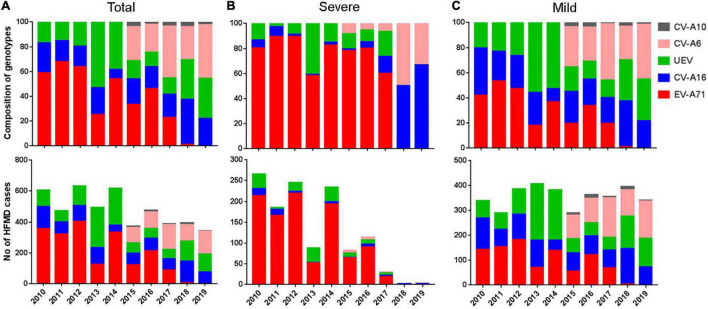
Enterovirus distribution of different serotypes in Nanchang, China from 2010–2019. **(A)** Number and composition of different serotypes in all laboratory confirmed cases. **(B)** Number and composition of different serotypes in mild cases. **(C)** Number and composition of different serotypes in severe cases. After 2015, typing of CV-A6 and CV-A10 was included.

From 2010 to 2012, EV-A71 was a major pathogen, accounting for 58.9–68.9% of EV-positive cases. However, other unspecified enteroviruses (UEV) accounted for 53.2%, followed by 24.7% of EV-A71 and 22.1% of CV-A16 in 2013. In the following years, similar pattern was observed in 2015 and 2017, while EV-A71 turned predominant in 2014 and 2016, accounting for 54.0 and 45.8%, respectively (Figure 4). In 2018, only 2 cases associated with EV-A71 infection, and for the first time in 2019, no EV-A71 positive cases were reported. Two coxsackieviruses, CV-A6 and CV-A10, have been monitored since 2015 according to the modified provincial guideline of surveillance network. The surveillance results showed that CV-A6 account for 27.6, 22.6, 41.7, 26.9, and 43.6% of EV-positive cases from 2015 to 2019, respectively, making it one of the major causative agents of HFMD (Figure 4A). This result suggested that CV-A6 might be prevalent in Nanchang earlier than 2015 when this virus was included for molecular typing. However, the proportion of CV-A10 in laboratory-confirmed cases ranged from 2.2 to 3.9% during 2015–2019.

During 2010–2016, EV-A71 accounted for 78.1–89.4% of severe HFMD cases, with the exception of 2013, when the composition of EV-A71 in severe cases was 58.0%. Notably, the proportion of EV-A71 infections decreased to 24.7% in 2013, while UEV reached to a peak (53.2%), and a similar pattern was also observed in 2015 and 2017 (Figure 4B). Since 2018, the rate of severe cases has sharply decreased to less than 1% due to few EV-A71 infections (Figure 4B). These results suggest that EV-A71 is significantly associated with the severity of HFMD, as shown by Pearson correlation analysis (*r* = 0.919, *p* < 0.001). CV-A16 occurred in 1.2–13.3% (median 4.3%) of severe cases during 2010–2017, indicating a slight effect of CV-A16 on the incidence of severe cases. During 2010–2014, EV-A71, CV-A16 and UEV accounted for 37.9, 25.6, and 36.5% mild cases, respectively (Figure 4C). During 2015–2019, CV-A6 accounted for 34.8% mild cases followed by CV-A16 (25.3%), UEV (23.2%), EV-A71 (14.1%), and CV-A10 (2.6%) (Figure 4C).

### Phylogenetic Analysis of Coxsackievirus A6

**FIGURE 5:**
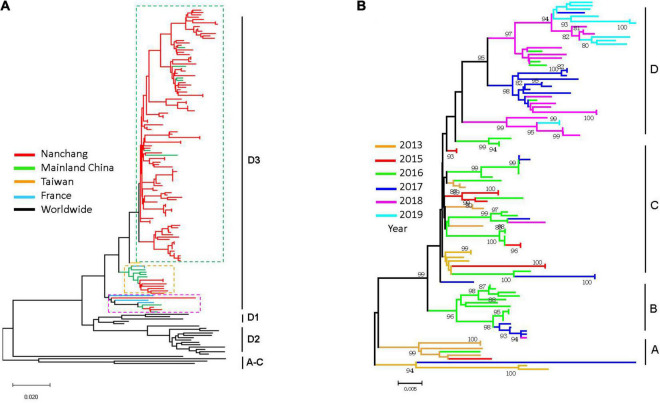
Phylogenetic analysis of VP1 sequences of CV-A6 strains circulating in Nanchang in 2013–2019 and representative CV-A6 strains worldwide. **(A)** Branches are colored by regions which include Nanchang strains indicated. **(B)** Phylogenetic tree of CV-A6 strains circulating in Nanchang during 2013–2019.

Phylogenetic analysis indicated that 106 representative strains obtained from 2013 to 2019 belonged to subgenotype D3, distributing in three major branches (Figure 5A), showing 89.7–99.9% identity for nucleotides and 98.3–100% identity for amino acids. We observed that some strains were similar to strains from France and strains from other cities in mainland China. However, most strains from Nanchang were clustered in a separate branch and all these representative strains circulating in Nanchang were distributed into 4 main clusters, as shown in Figure 5B. A temporal evolutionary pattern emerged from cluster A to D, where strains from 2013 were distributed in cluster A and C. In cluster B, most strains were obtained in 2016 and 2017. In cluster C, the strains identified during 2013–2018 merged with each other and evolved. In cluster D, some CV-A6 strains evolved to a new level and a subgroup of CV-A6 strains emerged in 2019. Strains circulating during 2013–2015 are no longer detected in cluster D.

### Age and Gender Distributions of Hand, Foot, and Mouth Disease

**FIGURE 6:**
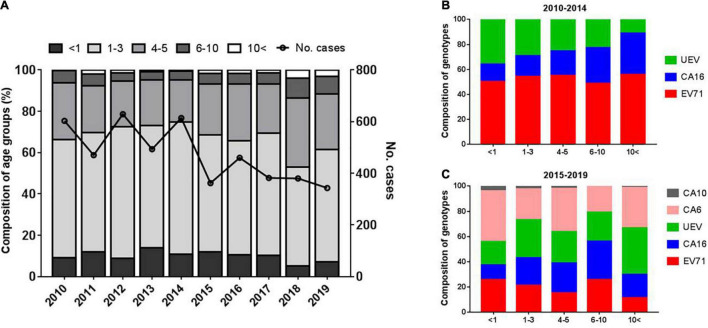
Characteristics of age distribution of HFMD cases **(A)** and enterovirus distribution of different serotypes in different age groups **(B,C)**.

To explore gender differences, we found male cases were 1.4–2.1 times more common than females. Despite the higher incidence of HFMD in males, there was no significant difference in the proportion of severe diseases between males and females. Children aged 0–3 years accounted for 67.5% of laboratory-confirmed cases, with an average of 9.9% of children under 1 year of age. Children aged 4–5 years accounted for 25.0%, while only 5.8% of cases occurred in children aged 6–10 years. Infections in people aged 10 years and older were rare, accounting for approximately 1.4% (Figure 6A). The results indicated HFMD generally affects children under 5 years of age and that children under 3 years of age were at the highest risk of enterovirus infection.

To explore the distribution of the pathogenic spectrum of enteroviruses across age groups, we found that CV-A16 ranged from 13.9 to 33.3% with an increasing trend with age from 2010 to 2014, while EV-A71 accounted for 50.0–55.0% cases in all age groups (Figure 6B). During 2015–2019, the proportion of EV-A71 ranged from 14.6 to 25.4%, with an overall trend of decreasing with age, while the proportion of CV-A16 increased with age, but with limited cases older than 10 years. The highest proportion of CV-A6 (40.7%) was in those younger than 1 year (Figure 6C).

### Seasonality of Enterovirus by Serotypes

**FIGURE 7:**
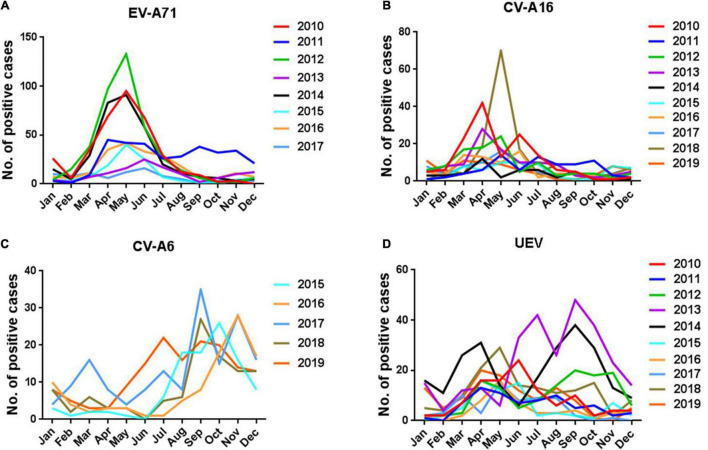
Seasonal features of enterovirus by serotypes. **(A)** EV-A71; **(B)** CV-A16; **(C)** CV-A6; **(D)** UEV.

The incidence of HFMD generally peaked in the second quarter of 2010, 2011, 2012, and 2014, and the monthly distribution of HFMD cases is shown in Figures 3A,C. To determine the seasonal characteristics of EV-A71, CV-A16, CV-A6, and UEV, we analyzed their monthly distribution. It was observed that the prevalence patterns of EV-A71 and CV-A16 were similar and both of them peaked from March to July and then leveled off (Figures 7A,B). However, CV-A6 was generally prevalent after July and peaked from August to November (Figure 7C). As for UEV, it had two peaks around April and September, throughout the year (Figure 7D).

### Impact of Enterovirus 71 Vaccination on the Pattern of Hand, Foot, and Mouth Disease Epidemic in Nanchang

The EV-A71 vaccine approved by the Chinese FDA has been available for children under 5 years of age in Nanchang since June 2016. As of 2016, a total of 3,034 children were vaccinated with EV-A71vaccine. In 2017, EV-A71 vaccination was classified as Category 2 vaccine in the National Immunization Program (NIP). A total of 55,632 children were vaccinated in 2017, with a significant decrease in the proportion of EV-A71 infections (Supplementary Figure 1). In 2018, the number of vaccinations (*n* = 102,145) almost doubled compared to the previous year, with only 2 cases of EV-A71-related cases. Later, EV-A71-related cases has not been reported since August 2018. In 2019, a total of 87,679 children were vaccinated as recorded, and about 26.4% of children under 3 years of age in Nanchang were vaccinated as of 2019 (Supplementary Figure 2). Correlation analysis indicated that the EV-A71 vaccination was negatively correlated with the incidence of EV-A71 infection (*r* = −0.990, *p* = 0.01). The results indicated EV-A71 vaccination is effective in containing this virus in Nanchang.

### The Influence of Enterovirus 71 Vaccination on the Incidence of Different Subtypes of Hand, Foot, and Mouth Disease

**FIGURE 8:**
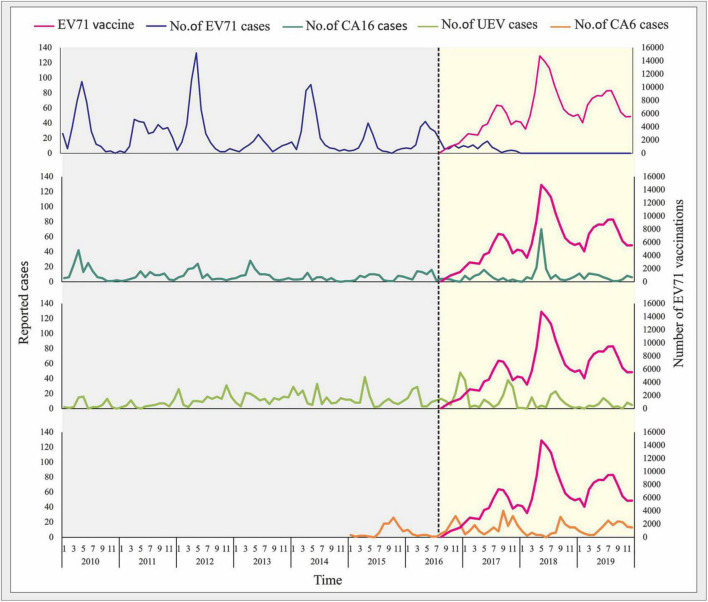
The influence of EV-A71 vaccination on the incidence of different subtypes of HFMD. Serotyping of CV-A6 was included since 2015, and EV-A71 vaccination was initiated since July 2016.

It can be seen from Figure 8 that after July 2016, the number of people vaccinated with EV-A71 in Nanchang City has increased its volatility. The number of EV-A71 cases before vaccination was the type with the highest proportion among the pathogenic subtypes of HFMD. After the vaccine was put into inoculation, the number of EV-A71 cases decreased significantly, while the number of cases caused by the other three subtypes did not show a significant downward trend.

## Discussion

From 2008–2012, EV-A71 was the major pathogen causing HFMD and was responsible for most severe and fatal cases in mainland China (Xing et al., 2014). In this study, we reported on the epidemiology of HFMD based on sentinel pathogenic surveillance in Nanchang, where different epidemiological patterns of HFMD were observed over time, especially when EV-A71 vaccine was planned as Category 2 vaccine since 2016.

From 2010 to 2012, EV-A71 dominated, accounting for 58.9–67.9% of EV-positive cases and 80.4–89.4% of severe cases. CV-A16 and UEV accounted for 15.3–19.8% and 16.6–23.9% of cases, respectively. Our previous studies have shown that locally circulating EV-A71 strains belong to the C4a sub-genotype, which is a dominant genotype in mainland China (Chen et al., 2014; Gao et al., 2014; Zhou et al., 2015; Ji T. et al., 2019). The proportion of UEV increased significantly and fluctuated during 2013–2016, the UEV proportion markedly increased and fluctuated, followed by a sharp decline in severe cases after the proportion of EV-A71 bottomed out in 2013 and 2015. This result indicated a positive correlation between the proportion of EV-A71 and the proportion of severe cases.

A nationwide (23 provinces) survey also indicated a dominance of UEV in 2013 and 2015, when it was clarified that the proportion of CV-A6 in UEV was 80 and 59% in 2013 and 2015, respectively (Li et al., 2018). Although that survey did not include data from Jiangxi Province, we observed here that the positive rate of CV-A6 among UEV cases in Nanchang ranged from 42.7 to 71.2% during 2015–2019. Another study conducted in Shanghai during 2012–2013 found that CV-A6 gradually became the dominant pathogen of HFMD from the end of 2012 (Wang et al., 2018). CV-A6 emergence in Shanghai coincided with its occurrence in southern Vietnam, where CV-A6 strains belong to a viral lineage consisting of CV-A6 strains from the United Kingdom and Asian countries, including China (Anh et al., 2018). Although the results of relative genetic diversity emphasize the global spread of CV-A6, phylogeographic analysis of global strains did not reveal a high frequency of CV-A6 movement between endemic countries.

In this study, we monitored two coxsackieviruses, CV-A6 and CV-A10, from an expanding number of UEV cases since 2015, and we observed that CV-A6 replaced EV-A71 as the predominant pathogen of HFMD (Supplementary Figure 1). CV-A10 accounts for approximately 3% of EV-positive cases per year, and it was rarely detected from severe cases throughout surveillance it was detected, indicating that it is less responsible for the severity of the disease. Phylogenetic analysis showed that CV-A10 strains of genotype B were mainly distributed in two branches, B1 and B2, intermingling with strains from other cities in China (Supplementary Figure 3A). And these circulating strains underwent cross-evolution in Nanchang after 2013 (Supplementary Figure 3B). Previous evidence suggests that CV-A6 began sporadically spreading in China from late 2012 before turning dominant in 2013 and 2015 (Feng et al., 2015; Song et al., 2017; Li et al., 2018; Wang et al., 2018). Despite the lack of publicly available CV-A6 surveillance data after 2015, our survey observed a dominant trend of CV-A6 in Nanchang. Although we could not see the whole picture of the epidemiological features of CV-A6 from the large proportion of UEV in 2013 and 2014, the seasonal distribution patterns provided us a speculation that CV-A6 dominated the UEV cases in both years. Although samples collected prior to 2014 were incompletely preserved, we performed retrospective testing for CV-A6 and CV-A10 on the remaining from 2013. Of the 148 UEV samples collected from June to September 2013, 89 (60.1%) were positive for CV-A6 and 5 (3.4%) were positive for CV-A10. The results confirmed that CV-A6 has been widely circulated in Nanchang since 2013.

In 2016, the EV-A71 vaccine approved by the Chinese FDA was firstly implemented in China and then programmed as Category 2 vaccine (Li et al., 2014). In this study, we observed that the proportion of EV-A71 continued to decrease as vaccination rates increased, at 22.5% in 2017, 0.5% in 2018 and disappeared from 2019, suggesting that immune protection in children is quite efficient after programmed EV-A71 vaccination. However, it is unclear whether cross-immunization occurs. It would be worthwhile to explore the seroepidemiology in healthy children in the near future. Apparently, EV-A71 vaccination has a twofold impact. On the one hand, it changes the pattern of pathogen spectrum and the distribution of different serotypes. On the other hand, it leaves us a new topic of close attention to the molecular and spatial epidemiology of CV-A6. Moreover, an in-depth exploration of the serotypes of other enteroviruses is important to understand the full picture of the etiology of HFMD, especially for those severe cases without serotypes. And the analysis of genetic variation and recombination of viruses will help us to better understand the genetic evolution of viruses, thus informing vaccine research and development. However, multicenter pathogenetic data on HFMD after 2016 are lacking for our understanding of the overall trend of EV-A71 in other regions of China, and differences in EV-A71 vaccine coverage in different regions could also affect the EV-A71 epidemic. Thus, laboratory surveillance of EV-A71 is still needed in the future, as this virus causes the most severe cases.

## Data Availability Statement

The original contributions presented in the study are included in the article/Supplementary Material, further inquiries can be directed to the corresponding author/s.

## Author Contributions

XZ and TC conceived and designed the study. FH, KQ, WX, JT, CZ, and XZ performed the experiments. FH, JR, YZ, QZ, SC, and XZ collected the data. FH, JR, ZD, SY, TC, and XZ analyzed and interpreted the data. XZ wrote the manuscript. All authors approved the manuscript.

## Conflict of Interest

The authors declare that the research was conducted in the absence of any commercial or financial relationships that could be construed as a potential conflict of interest.

## Publisher’s Note

All claims expressed in this article are solely those of the authors and do not necessarily represent those of their affiliated organizations, or those of the publisher, the editors and the reviewers. Any product that may be evaluated in this article, or claim that may be made by its manufacturer, is not guaranteed or endorsed by the publisher.

## References

[B1] Anh N. T. Nhu L. N. T. Van H. M. T. Hong N. T. T. Thanh T. T. Hang V. T. T. (2018). Emerging coxsackievirus A6 causing hand, foot and mouth disease, Vietnam. *Emerg. Infect. Dis.* 24 654–662. 10.3201/eid2404.171298 29553326 PMC5875260

[B2] Chen J. F. Zhang R. S. Ou X. H. Chen F. M. Sun B. C. (2014). The role of enterovirus 71 and coxsackievirus A strains in a large outbreak of hand, foot, and mouth disease in 2012 in Changsha, China. *Int. J. Infect. Dis.* 28 17–25. 10.1016/j.ijid.2014.07.024 25236389

[B3] Feng X. Guan W. Guo Y. Yu H. Zhang X. Cheng R. (2015). A novel recombinant lineage’s contribution to the outbreak of coxsackievirus A6-associated hand, foot and mouth disease in Shanghai, China, 2012-2013. *Sci. Rep.* 5:11700. 10.1038/srep11700 26121916 PMC4485158

[B4] Gao L. D. Hu S. X. Zhang H. Luo K. W. Liu Y. Z. Xu Q. H. (2014). Correlation analysis of EV71 detection and case severity in hand, foot, and mouth disease in the Hunan Province of China. *PLoS One* 9:e100003. 10.1371/journal.pone.0100003 24941257 PMC4062471

[B5] Horsten H. H. Kemp M. Fischer T. K. Lindahl K. H. Bygum A. (2018). Atypical hand, foot, and mouth disease caused by coxsackievirus A6 in Denmark: a diagnostic mimicker. *Acta Derm Venereol.* 98 350–354. 10.2340/00015555-2853 29182793

[B6] Ji H. Fan H. Lu P. X. Zhang X. F. Ai J. Shi C. (2019). Surveillance for severe hand, foot, and mouth disease from 2009 to 2015 in Jiangsu province: epidemiology, etiology, and disease burden. *BMC Infect. Dis.* 19:79. 10.1186/s12879-018-3659-7 30669973 PMC6341624

[B7] Ji T. Han T. Tan X. Zhu S. Yan D. Yang Q. (2019). Surveillance, epidemiology, and pathogen spectrum of hand, foot, and mouth disease in mainland of China from 2008 to 2017. *Biosafety Health.* 1 32–40.

[B8] Li R. Liu L. Mo Z. Wang X. Xia J. Liang Z. (2014). An inactivated enterovirus 71 vaccine in healthy children. *N. Engl. J. Med.* 370 829–837. 24571755 10.1056/NEJMoa1303224

[B9] Li Y. Chang Z. Wu P. Liao Q. Liu F. Zheng Y. (2018). Emerging enteroviruses causing hand, foot and mouth disease, China, 2010-2016. *Emerg. Infect. Dis.* 24 1902–1906. 10.3201/eid2410.171953 30226172 PMC6154135

[B10] Liu M. Y. Liu W. Luo J. Liu Y. Zhu Y. Berman H. (2011). Characterization of an outbreak of hand, foot, and mouth disease in Nanchang, China in 2010. *PLoS One* 6:e25287. 10.1371/journal.pone.0025287 21980416 PMC3182205

[B11] Liu Y. Fu C. Wu S. Chen X. Shi Y. Zhou B. (2014). A novel finding for enterovirus virulence from the capsid protein VP1 of EV71 circulating in mainland China. *Virus Genes* 48 260–272. 10.1007/s11262-014-1035-2 24442718

[B12] Palacios G. Oberste M. S. (2005). Enteroviruses as agents of emerging infectious diseases. *J. Neurovirol.* 11 424–433. 10.1080/13550280591002531 16287683

[B13] Shen X. X. Qiu F. Z. Zhao H. L. Yang M. J. Hong L. Xu S. T. (2018). A novel and highly sensitive real-time nested RT-PCR assay in a single closed tube for detection of enterovirus. *Diagn. Microbiol. Infect. Dis.* 90 181–185. 10.1016/j.diagmicrobio.2017.11.015 29273481

[B14] Solomon T. Lewthwaite P. Perera D. Cardosa M. J. McMinn P. Ooi M. H. (2010). Virology, epidemiology, pathogenesis, and control of enterovirus 71. *Lancet Infect. Dis.* 10 778–790. 10.1016/S1473-3099(10)70194-8 20961813

[B15] Song Y. Zhang Y. Ji T. Gu X. Yang Q. Zhu S. (2017). Persistent circulation of Coxsackievirus A6 of genotype D3 in mainland of China between 2008 and 2015. *Sci. Rep.* 7:5491. 10.1038/s41598-017-05618-0 28710474 PMC5511160

[B16] Tan X. Li L. Zhang B. Jorba J. Su X. Ji T. (2015). Molecular epidemiology of coxsackievirus A6 associated with outbreaks of hand, foot, and mouth disease in Tianjin, China, in 2013. *Arch. Virol.* 160 1097–1104. 10.1007/s00705-015-2340-3 25680566 PMC4629805

[B17] Wang J. Teng Z. Cui X. Li C. Pan H. Zheng Y. (2018). Epidemiological and serological surveillance of hand-foot-and-mouth disease in Shanghai, China, 2012-2016. *Emerg. Microbes Infect.* 7:8. 10.1038/s41426-017-0011-z 29362406 PMC5837173

[B18] Wang S. M. Liu C. C. Tseng H. W. Wang J. R. Huang C. C. Chen Y. J. (1999). Clinical spectrum of enterovirus 71 infection in children in southern Taiwan, with an emphasis on neurological complications. *Clin. Infect. Dis.* 29 184–190. 10.1086/520149 10433583

[B19] Xing W. Liao Q. Viboud C. Zhang J. Sun J. Wu J. T. (2014). Hand, foot, and mouth disease in China, 2008–12: an epidemiological study. *Lancet Infect. Dis.* 14 308–318. 10.1016/S1473-3099(13)70342-6 24485991 PMC4035015

[B20] Yin X. G. Yi H. X. Shu J. Wang X. J. Wu X. J. Yu L. H. (2014). Clinical and epidemiological characteristics of adult hand, foot, and mouth disease in northern Zhejiang, China, May 2008-November 2013. *BMC Infect. Dis.* 14:251. 10.1186/1471-2334-14-251 24885052 PMC4026826

[B21] Zhou X. Zhu Q. Xia W. He F. Hu M. Ni X. (2015). Molecular epidemiology of an outbreak of hand, foot, and mouth disease associated with subgenotype C4a of enterovirus A71 in Nanchang, China in 2014. *J. Med. Virol.* 87 2154–2158. 10.1002/jmv.24288 26058813

[B22] Zong W. He Y. Yu S. Yang H. Xian H. Liao Y. (2011). Molecular phylogeny of Coxsackievirus A16 in Shenzhen, China, from 2005 to 2009. *J. Clin. Microbiol.* 49 1659–1661. 10.1128/JCM.00010-11 21325543 PMC3122795

