# Supplementary material for: Surveillance, Epidemiology and Impact of EV-A71 Vaccination on Hand, Foot, and Mouth Disease in Nanchang, China, 2010–2019
Source: Front Microbiol. 2022 Jan 6;12:811553. doi: 10.3389/fmicb.2021.811553 (PMC8770912; doi:10.3389/fmicb.2021.811553)
Supplement: Supplementary file 1 [file Table_1.pdf]

Supplementary Table 1. Strains used for molecular analysis of CV-A6

Isolation year	Countries	Strain name	GenBank accession No.	Genotype/ Sub-genotype	Origin
1949	USA	Gdula	AY421764	A	GenBank
2004	China	AFP560/GD/CHN/2004	KP143074	B	GenBank
2005	China	AFP262/GD/CHN/2004	KP143075	B	GenBank
1996	China	96188/SD/CHN/1996	JQ464887	C	GenBank
1999	Japan	Kyoto1	AB779614	D1	GenBank
1999	Japan	Hyogo1278	LC126143	D1	GenBank
2008	Spain	ESP08/1023	FR797988	D1	GenBank
2010	France	CF165026FRA	HE572917	D1	GenBank
2010	France	CF181032FRA	HE572935	D1	GenBank
2009	China	JB143090122	KC866916	D2	GenBank
2009	China	JB143090119	KC866921	D2	GenBank
2009	Japan	Kyoto4	AB779617	D2	GenBank
2010	China	10032/SD/CHN/2020	JQ364889	D2	GenBank
2010	China	CV6-SHZH2010-0601	JX154921	D2	GenBank
2011	China	HN421/HN/CHN/2011	JN797598	D2	GenBank
2011	China	CVA6-SHZH2011-0507	JX473340	D2	GenBank
2011	China	SHAPHCI1883/SH/CHN/2011	JX495130	D2	GenBank
2011	China	075/GS/CHN/2011	KY211690	D2	GenBank
2013	China	A023/YN/CHN/2013	KY424358	D2	GenBank
2013	China	13-97/JL/CHN/2013	KY424390	D2	GenBank
2011	Japan	Shizuoka 1	AB649286	D3	GenBank
2008	Spain	ESP08/54694	FR79984	D3	GenBank
2010	China	10MF66 Q4 JS	KJ577275	D3	GenBank
2010	China	CVA6/SHZH2010-0905	JX154931	D3	GenBank
2010	China	10MF67 JS/CHN/2010	KJ577276	D3	GenBank
2010	France	CF140007FRA	HE572906	D3	GenBank
2010	France	CF175076FRA	HE572928	D3	GenBank
2010	France	CF194028FRA	HE572938	D3	GenBank
2010	China	TW/409/2010	JQ946055	D3	GenBank
2011	China	CVA6/SHZH2011-0701	JX473370	D3	GenBank
2013	China	13-87/SaX/CHN/2013	KY424394	D3	GenBank
2013	China	13-28/ZJ/CHN/2013	KY424409	D3	GenBank
2013	China	TJ13-54-C/CHN/2013	KJ848308	D3	GenBank
2014	China	14-106/JX/CHN/2014	KY424385	D3	GenBank
2014	China	14-106/HuN/CHN/2014	KY424386	D3	GenBank
2015	China	SHAPHC5896/SH/CHN/2015	KU736939	D3	GenBank
2017	China	2017-CA6-482	MG385817	D3	GenBank
2017	China	538/GD/CH/2017	MG385818	D3	GenBank
2017	China	60/GD/CH/2017	MG385819	D3	GenBank

Isolation year	Countries	Strain name	GenBank accession No.	Genotype/ Sub-genotype	Origin
2017	China	91/GD/CHN/2017	MG385831	D3	GenBank
2017	China	703/GD/CHN/2017	MG385829	D3	GenBank
2017	China	482/GD/CH/2017	MG385817	D3	GenBank
2013, 2015-2019	China	106 representative CV-A6 strains identified in Nanchang	MW075633-MW075642; OL677506-OL677510; OL688664-OL688754	D3	This study
