# Supplementary material for: Surveillance, Epidemiology and Impact of EV-A71 Vaccination on Hand, Foot, and Mouth Disease in Nanchang, China, 2010–2019
Source: Front Microbiol. 2022 Jan 6;12:811553. doi: 10.3389/fmicb.2021.811553 (PMC8770912; doi:10.3389/fmicb.2021.811553)

Supplementary Figure 1

Proportion of enterovirus serotypes in different period during 2010-2019 in Nanchang city.

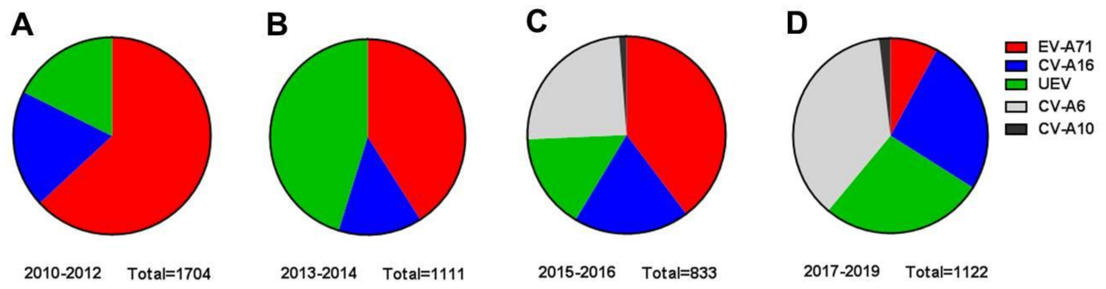

Supplementary Figure 2

Number of children (<3 years) population and EV-A71vaccination rate during 2016-2019 in Nanchang city.

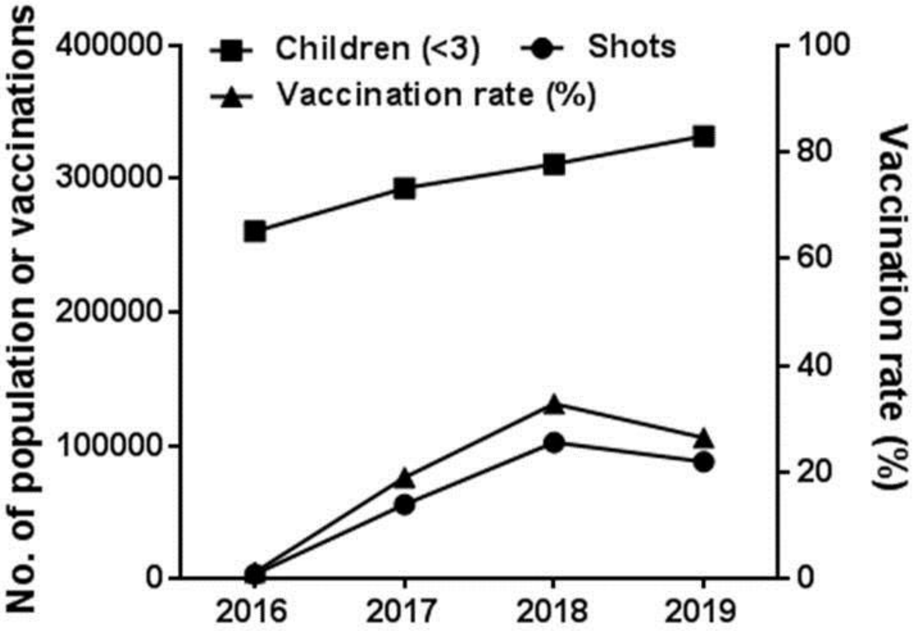

Supplementary Figure 3

Phylogenetic analysis of VP1 sequences of CV-A10 strains circulating in Nanchang during 2013-2019.

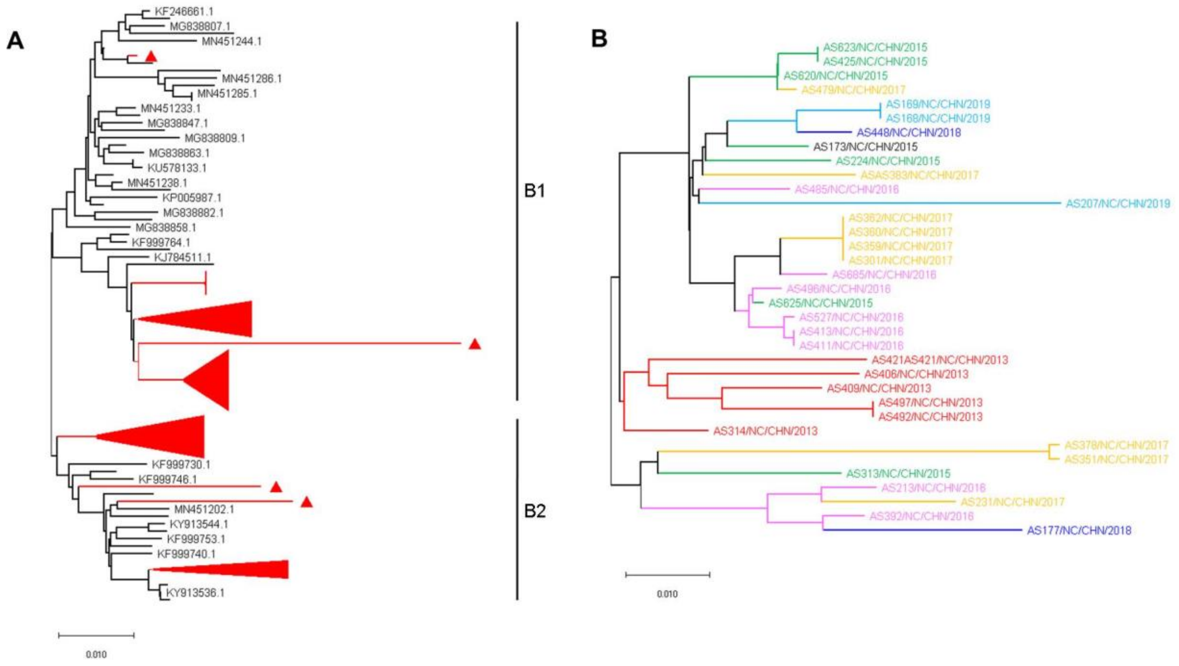

Supplement: Supplementary file 2 [file Presentation_1.pdf]
